# Seed Pretreatment and Foliar Application of Proline Regulate Morphological, Physio-Biochemical Processes and Activity of Antioxidant Enzymes in Plants of Two Cultivars of Quinoa (*Chenopodium quinoa* Willd.)

**DOI:** 10.3390/plants8120588

**Published:** 2019-12-10

**Authors:** Hira Yaqoob, Nudrat A. Akram, Samrah Iftikhar, Muhammad Ashraf, Noman Khalid, Muhammad Sadiq, Mohammed Nasser Alyemeni, Leonard Wijaya, Parvaiz Ahmad

**Affiliations:** 1 Department of Botany, Government College University, Faisalabad 38040, Pakistan; hira.yaqoob@gmail.com (H.Y.); samrahiftikhar@gmail.com (S.I.); nomankhalid@gmail.com (N.K.); sadiq.muhammad@gmail.com (M.S.); 2 University of Agriculture, Faisalabad 38040, Pakistan; ashrafbot@yahoo.com; 3 Botany and Microbiology Department, College of Science, King Saud University, Riyadh 11451, Saudi Arabia; mnyemeni@ksu.edu.sa (M.N.A.); leon077@gmail.com (L.W.); 4 Department of Botany, S.P. College, Srinagar 190001, Jammu and Kashmir, India

**Keywords:** antioxidants, low temperature stress, proline, Quinoa (*Chenopodium quinoa*)

## Abstract

In the current study, the effects of exogenously applied proline (25 and 50 mM) and low-temperature treatment were examined on the physiochemical parameters in the plants of two cultivars (V_1_ and V_2_) of quinoa (*Chenopodium quinoa* Willd.). The seeds were also exposed to chilling stress at 4 °C before sowing. Plants raised from the seeds treated with low temperature showed reduced plant growth and contents of chlorophyll and carotenoids, but they had significantly increased contents of malondialdehyde, proline, ascorbic acid, total free amino acids, total soluble sugars, and total phenolics, as well as the activity of the peroxidase (POD) enzyme. Cold stress applied to seeds remained almost ineffective in terms of bringing about changes in plant root, hydrogen peroxide, glycine betaine and activities of superoxide dismutase (SOD), and catalase (CAT) enzymes. The exogenous application of proline significantly increased plant growth, the contents of chlorophyll, carotenoids, proline, ascorbic acid, total free amino acids, phenolics, and total soluble sugars, as well as the activities of SOD, POD, and CAT, but it decreased malondialdehyde content. Overall, foliar application of proline was better than the seed treatment in improving root dry weight, root length, chlorophyll *a*, carotenoids, glycine betaine, ascorbic acid and superoxide dismutase activity, whereas seed pre-treatment with proline was effective in improving shoot dry weight, shoot length, hydrogen peroxide, malondialdehyde, and peroxidase activity in both quinoa cultivars.

## 1. Introduction

Abiotic factors include cold, heat, drought, salinity, toxicity of heavy metals, etc. that severely influence plant growth and development, thereby decreasing overall plant yield. They also act as growth-limiting factors by producing reactive oxygen species (ROS), ultimately causing oxidative stress [1,2,3]. This oxidative stress triggers metabolic disorders and causes a loss of organelle functions and cell injury, thereby leading to cell death [4,5]. Cold stress is one of the major abiotic stresses that directly influences as well as reduces plant growth and yield [6,7,8]. It has been estimated that up to 25% of crop yield losses occur due to chilling stress [9,10]. To cope with unfavorable situations, plants have evolved molecular as well as physio-biochemical adaptations [11]. In response to chilling stress, a number of defensive processes activate within the cells such as cell structure modification, accumulation of antioxidants and osmoprotectants, modification in gene expression, and the generation of anti-freezing proteins [12,13].

Under abiotic stresses, osmoprotectants such as sugars, polyols and proline accumulate which can effectively enhance stress tolerance in plants. They can be utilized as molecular markers for breeding for enhanced stress tolerance [14,15]. Of a variety of osmoprotectants, proline is considered as a vital biomolecule and has been reported to play a significant role in various stresses, both abiotic and biotic [16]. Proline also acts as a potential scavenger of reactive oxygen species [17]. For maintaining optimal cellular levels of proline, foliar application of proline is an effective option [18].

Proline also facilitates the transduction of signals, acts as a substitute of carbon and nitrogen, decreases the membranous injury carried out by ROS species, and maintains protein complexes and DNA, thus performing a number of imperative functions in plants subjected to stressful cues [19]. Stress resistance in plants also increases by the exogenous application of proline, which acts as a cryo-protectant or osmoprotectant [20].

Under stress conditions, proline constitutes approximately 80% of free amino acids in plants [21]. Proline not only participates in the synthesis of primary metabolites, but it also transports metabolites at the point of growth and development [22,23]. Proline is believed to help in oxidative phosphorylation in mitochondria and triggers ATP synthesis as a stress recovery mechanism [24]. Proline shows stress ameliorative effects in different crops; e.g., maize [25], tobacco [26], wheat [27], and olive [28]. 

Of various cereal crops, *Chenopodium quinoa* has an outstanding food quality as well as high resistance to abiotic stresses [29,30]. Due to this fact, the cultivation of this crop has spanned the globe [31,32]. It can be cultivated in the Andean regions with nutritionally poor lands, and native people there use it as a staple food [32,33]. Seeds of quinoa are a rich source of minerals (magnesium, zinc, lithium, copper, iron and calcium), vitamins (A, B_2_ and E), basic amino acids, fatty acids and carbohydrates [34,35], which are medicinally very important [29]. Seeds are gluten free and can be used to prepare gluten-free food for celiac patients [29]. 

We hypothesized that the exogenous application of proline or low temperature could effectively improve key morpho-physiological and biochemical attributes in *Chenopodium quinoa* plants, which in turn could improve growth. Thus, in the present study, the role of proline and low temperature as a pre-sowing seed treatment and proline foliar application in promoting growth, some biochemical processes, and the oxidative defense system were assessed in quinoa plants.

## 2. Materials and Methods

### 2.1. Experimental Design and Treatment

The research was carried out to investigate the effect of proline applied as a seed treatment and through leaves to quinoa (*Chenopodium quinoa*) plants raised from seeds treated with low-temperature stress. An experiment was conducted under natural climatic conditions during October–December, 2016 at the Department of Botany, GC University Faisalabad, Pakistan. Seeds of two different quinoa cultivars, namely V_1_ and V_2_ (with different genetic makeup), were provided by the Crop Physiology Department, University of Agriculture, Faisalabad, Pakistan. For cold treatment, the seeds were taken in Petri plates and kept at 4 °C in a refrigerator for 20 h to induce chilling stress. After that, the seeds were treated with varying effective concentrations (0, 25 and 50 mM) of proline for 15 h following Kamran et al. [36]. The seeds so primed were sown in disposable plastic glasses each containing 0.407 kg sandy loam soil. The experiment was arranged in a completely randomized design with three replicates per treatment. Thinning was done after three weeks of germination to maintain five plants of equal size per pot. During the course of the experiment, the average temperature of 28 °C (day) and 20 °C (night), relative humidity of 61% and day-length of 7.5 h were recorded. After one month of seed germination, proline was applied as a foliar spray using an ordinary sprayer with a sharp nozzle. Three concentrations (0, 25 and 50 mM) of proline were prepared in distilled water containing 0.1% Tween-20 as a surfactant. To the chilled but non-primed seedlings, proline was applied as a foliar spray. After two weeks of exogenous application, the plant samples were collected to measure the following parameters.

### 2.2. Plant Growth

Two plants were harvested from each replication at 40 days after seed germination and washed carefully with distilled water to dislodge the soil particles. The fresh weights of all shoot and root samples were precisely measured; then, all samples were placed in an oven at 80 °C for 72 h, and then the dry weights were recorded.

### 2.3. Chlorophyll Contents

Fresh leaf material (each 0.5 g) was extracted with 80% acetone in a pestle and mortar followed by centrifugation to obtain the supernatant [37]. The optical density of the supernatant was read at 645 and 663 nm using a spectrophotometer. 

### 2.4. Hydrogen Peroxide (H_2_O_2_) Contents

The leaf material (0.25 g) was triturated using a pestle and mortar containing 5 mL of 0.1% trichloroacetic acid following the method of Velikova, et al. [38]. The homogenized material was centrifuged for 15 min at 12,000× *g*. To 0.5 mL of the supernatant, 1 mL of 1 M potassium iodide and 0.5 mL of potassium phosphate buffer were added, and the mixture was shaken vigorously. The absorbance of the mixture was read at 390 nm using a spectrophotometer.

### 2.5. Malondialdehyde (MDA) Contents

The protocol of Heath and Packer [39] was employed to determine the contents of MDA. Fresh leaf material (each 0.25 g) was ground with 2.5 mL of 5% (*w*/*v*) trichloroacetic acid (TCA). The mixture was centrifuged at 12,000× *g* for 15 min. Then, 1 mL of the supernatant and 4 mL of thiobarbituric acid (TBA) were mixed. The mixture was placed in a water bath at 95 °C for 30 min, the reaction mixture was chilled, and the absorbance was read at 532 and 600 nm using a spectrophotometer.

### 2.6. Free Proline Contents

The leaf samples (each 0.25 g) were ground each in 5 mL of sulfosalicylic acid following the method of Bates, et al. [40]. To 2.0 mL of plant extract, 2 mL of glacial acetic acid and 2.0 mL of acidic ninhydrin (2 mL) were added. The mixture was shaken vigorously and placed in a water bath for one h at 75 °C. To stop the reaction, the mixture was placed on ice. The proline contents were obtained from the chromophore layer by adding 4 mL of toluene to the reaction mixture. The toluene containing the chromophore was isolated and the absorbance read at 520 nm.

### 2.7. Ascorbic Acid

The Mukherjee and Choudhuri [41] protocol was employed to determine the contents of ascorbic acid. Plant material was homogenized with 6% TCA solution and filtered. An aliquot (2 mL) of the filtrate was mixed with 1 mL dinitrophenyl hydrazine in 9N H_2_SO_4_ (2%), and then a droplet of thiourea (prepared in 70% ethanol) was added to it. The samples were placed in a water bath for 15 min and then cooled at room temperature. After that, 2.5 mL of 80% sulfuric acid was added to the reaction mixture and the optical density (OD)was measured at 530 nm using a spectrophotometer.

### 2.8. Antioxidant Enzymes

To obtain plant crude extract, fresh ice-cold leaves (each 0.25 g) were homogenized in 10 mL of 50 mM of potassium phosphate buffer; then, the mixture was centrifuged and the supernatant preserved in Eppendorf tubes in a freezer to appraise the activities of the following enzymes.

### 2.9. Catalase (CAT) and Peroxidase (POD)

The activities of CAT and POD enzymes were determined following the protocol of Chance and Maehly [42]. Crude enzyme extract (100 μL), 1.9 mL of 50 mM buffer (1 mL) and 1 mL of H_2_O_2_ (40 mM) were mixed. The variation in absorbance of the reaction solution was read subsequently after every 20 seconds for 3 min at 240 nm using a spectrophotometer. To determine the peroxidase activity, the reaction mixture contained distilled water, 250 μL phosphate buffer (50 mM), 100 μL H_2_O_2_, 20 mM guaicol (100 μL) and the enzyme extract (50 μL). The change in the enzyme activity was noted after every 20 seconds for 3 min at 470 nm using a spectrophotometer. One unit of CAT and POD activity was defined as 0.01 absorbance change per min.

### 2.10. Superoxide Dismutase (SOD)

The protocol of Giannopolitis and Ries [43] was followed to determine the SOD activity. The reaction mixture for the SOD had 50 mM phosphate buffer, 13 mM methionine, 1.3 μM riboflavin, distilled water, and 50 μL supernatant. The reaction mixture was placed under a fluorescent lamp for 15 min and the absorbance read at 560 nm using a spectrophotometer. One unit of SOD activity was defined as the amount required to inhibit the photo-reduction of nitroblue tetrazolium (NBT) by 50%. 

### 2.11. Total Soluble Sugars

For the estimation of total soluble sugars, leaf material (each 0.5 g) was ground with 5 mL of 80% ethanol in a pestle and mortar. The samples were subjected to shaking for 1 h at 60 °C. To one mL of the supernatant, 3 mL of anthrone reagent (dissolved 150 mg anthrone in 72% freshly prepared H_2_SO_4_) was added; then, the mixture was heated for 10 min, the samples cooled down at room temperature for 20 min, and the absorbance read at 625 nm using a spectrophotometer following Yemm and Willis [44].

### 2.12. Total Phenolics

Leaf samples (each 0.5 g) were homogenized, each in 10 mL of 80% acetone. The mixture was centrifuged at 15,000× *g* at 4 °C for15 min. To 0.1 mL of plant extract, 2 mL of distilled water and 0.5 mL of Folin–Ciocalteau’s reagent were added. The samples were then shaken vigorously. By adding 2.5 mL of 20% sodium carbonate to each sample, all samples were vortexed for 10 seconds and their final volume was raised to 7.5 mL by adding distilled water. After 20 min, the absorbance of all samples was read at 750 nm following Julkunen-Tiitto [45]. 

### 2.13. Free Amino Acids

The leaf sample (each 0.5 g) was ground in 10 mL of potassium phosphate buffer. The mixture was centrifuged at 12,000× *g* at 4 °C. To 1 mL of the supernatant, 1 mL of ninhydrin (2%) and 1 mL of pyridine (10%) were added, and the mixture was placed in a water bath for 30 min. After cooling all samples, they were read at 750 nm using a spectrophotometer as described by Hamilton, et al. [46]. 

### 2.14. Statistical Analysis

The experiment was arranged in a completely randomized design with three factors (cultivars, cold stress, exogenous application) with three replicates. After working out the analysis of variance of data for each parameter, the means were compared at a 0.05% level with the least significant difference (LSD) test.

## 3. Results

**Figure 1:**
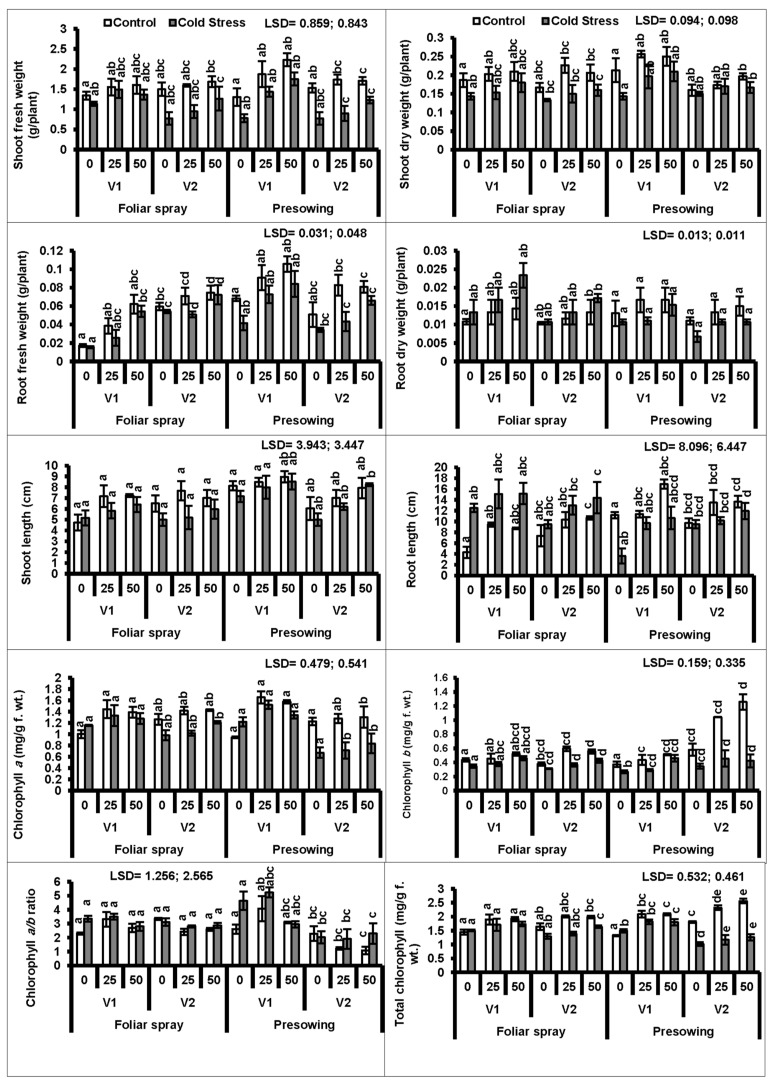
Shoot fresh and dry weights, root fresh and dry weights, shoot and root length, chlorophyll pigments (a and b), chlorophyll a/b ratio and total chlorophyll of quinoa plants subjected to chilling stress (mean ± S.E.); Different letters (a–c) show significant difference among treatments. f. wt; fresh weight.

Seeds of two quinoa cultivars (V_1_ and V_2_) treated with cold stress at 4 °C for 20 h were grown for 40 days under natural growth conditions (Supplementary Figure S1). Data showed that the shoot fresh and dry weights of the plants of both quinoa cultivars raised from cold-stressed seeds were significantly (*P* ≤ 0.001) reduced as compared to those of the plants raised from non-treated seeds. An exogenous application (pre-sowing) of proline (25 and 50 mM) was useful in improving the shoot fresh as well as dry weights of both quinoa cultivars under stress and non-stress conditions. The cultivar V_1_ had greater shoot fresh and dry weights than those of V_2_ under cold stress (Figure 1, Table 1 and Table 2).

Plants of both cultivars raised from the seeds treated with cold stress had significantly lower root fresh and dry weights than those of the plants raised from non-treated seeds. Both modes of application (foliar and seed pre-treatment) of proline showed a significant (*P* ≤ 0.01) enhancing effect only on the root fresh weight. Of both levels of proline, 50 mM proved the most beneficial. The cultivar V_1_ showed better performance with pre-sowing seed treatments in terms of the root fresh weight (Figure 1).

Plants raised from cold-stressed seeds showed significantly reduced shoot and root lengths (*P* ≤ 0.001; 0.05) in both quinoa varieties. However, proline applied via foliar and seed pre-treatment significantly (*P* ≤ 0.01; 0.001) increased only the root length. The response of quinoa cultivar V_1_ was more prominent with 50 mM proline applied as a seed pretreatment. 

A considerable reduction was observed in chlorophyll *a*, chlorophyll *b*, and the total chlorophyll in the leaves of plants of both quinoa cultivars (V_1_ and V_2_) rose from cold-stressed seeds. Externally applied (foliar and pre-sowing seed treatment) proline significantly increased both chlorophyll *a* and *b* in both quinoa cultivars. In the case of chlorophyll *a* and *b* contents, both proline concentrations (25 and 50 mM) as seed treatments were more effective in cultivar V_1_ compared to V_2_ (Figure 1).

**Figure 2:**
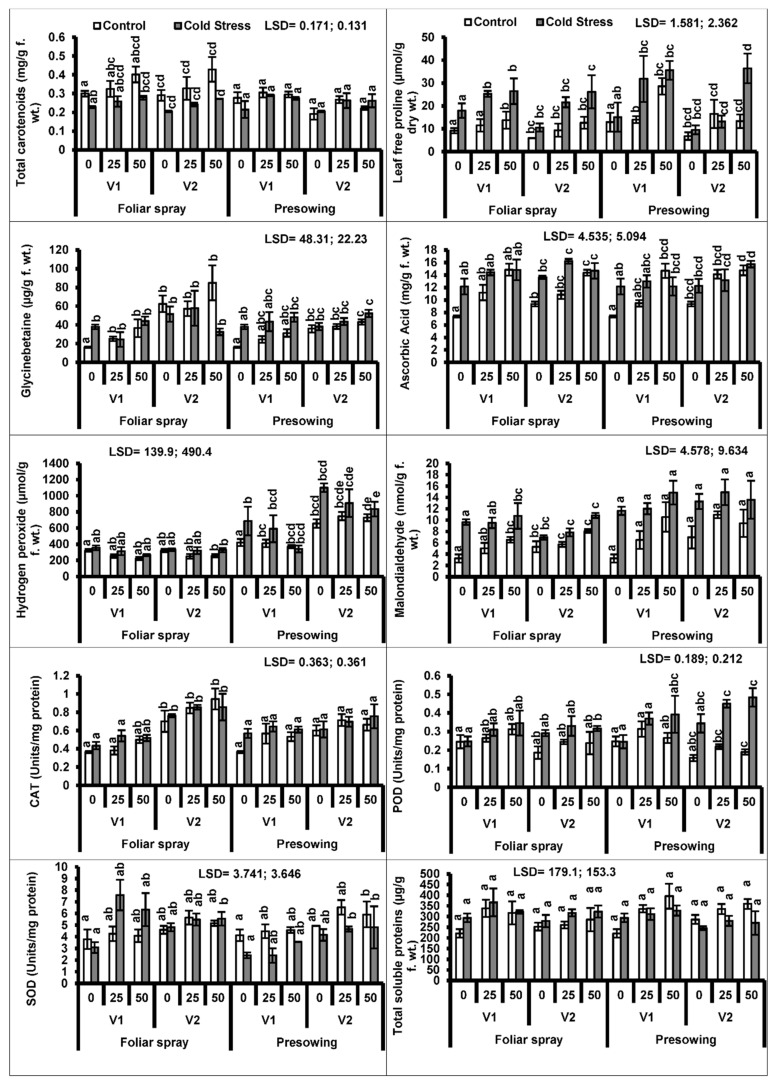
Total carotenoids, proline, glycine betaine, ascorbic acid, hydrogen peroxide, malondialdehyde contents, activities of enzymatic antioxidants (catalase (CAT), peroxidase (POD) and SOD) and total soluble proteins of quinoa plants subjected to chilling stress (mean ± S.E.); Different letters (a–e) show significant differences among treatments. f. wt; fresh weight.

A prominent reduction (*P* ≤ 0.001) occurred in carotenoid contents, particularly in quinoa plants raised from cold-stressed seeds. The exogenous application (foliar and pre-sowing) of proline considerably (*P* ≤ 0.01) promoted carotenoid contents in both quinoa cultivars. A similar trend was observed in both quinoa cultivars under both types of treatments (Figure 2, Table 1 and Table 2).

Low-temperature stress imposed as a pre-sowing treatment caused an enhanced accumulation of endogenous proline in the quinoa plants. The exogenous application of proline significantly (*P* ≤ 0.001) improved the in vivo proline levels in the plants of both cultivars raised from chilling-stressed seeds. The proline applied as a foliar spray was most effective in enhancing endogenous proline levels in both quinoa cultivars (Figure 2).

Intrinsic glycine betaine (GB) contents increased in plants raised from low-temperature stressed seeds. However, seed treatment with proline was effective in enhancing intrinsic GB contents only in cultivar V_2_ (Figure 2).

Ascorbic acid contents increased considerably in plants of both quinoa cultivars raised from cold-stressed seeds (Table 1 and Table 2). Both modes of proline application were effective in promoting AsA concentration in quinoa plants. Both quinoa cultivars showed a similar trend of increasing AsA contents under plants raised from seeds subjected to low temperature (Figure 2).

The quinoa plants raised from cold-treated seeds showed a significant increasing effect on the accumulation of hydrogen peroxide (H_2_O_2_) contents. Only the foliage spray of proline application played a significant role in lowering the H_2_O_2_ levels in the quinoa plants (Figure 2).

A high accumulation of malondialdehyde (MDA) was observed in the plants of both quinoa cultivars raised from low-temperature treated seeds. However, the exogenous application of proline through foliar spray (*P* ≤ 0.001) increased the MDA levels in both quinoa cultivars under chilling stress (Figure 2).

The activity of peroxidase enzyme significantly increased (*P* ≤ 0.001), while the activities of catalase (CAT) and superoxide dismutase (SOD) enzymes remained unaffected in quinoa plants developed from seeds treated with cold. The activities of CAT and SOD enzymes were better in quinoa cultivars due to the foliar-applied proline, whereas only the peroxidase activity of both cultivars was affected by the pre-sowing treatment (Figure 2).

**Figure 3:**
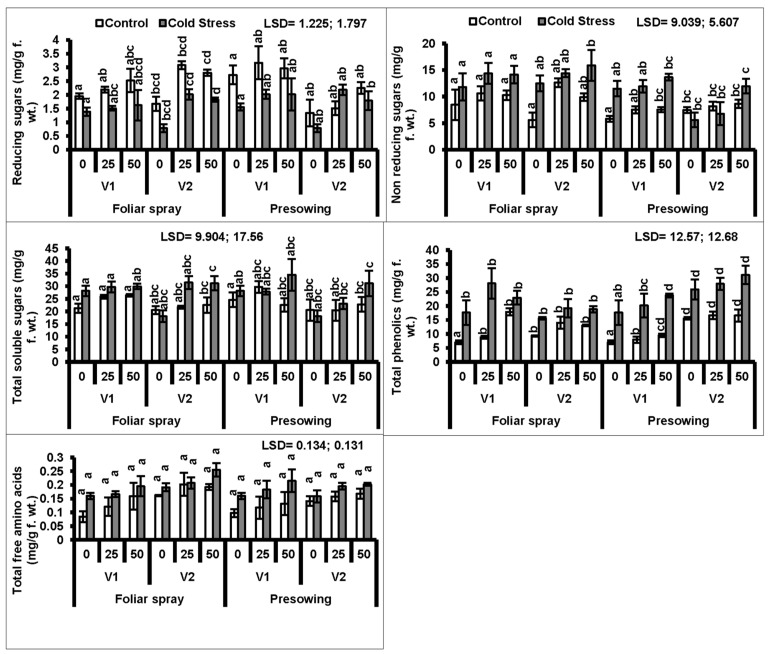
Reducing sugars, non-reducing sugars, total soluble sugars, total phenolics and total free amino acids of quinoa plants subjected to chilling stress (mean ± S.E.); Different letters (a–d) show significant differences among treatments. f. wt; fresh weight.

In the present study, cold stress applied as a seed pre-treatment significantly (*P* ≤ 0.001) increased the total phenolics in both cultivars of quinoa. The exogenous application of proline as a foliar spray only further increased the total phenolic contents (Figure 3, Table 1 and Table 2).

A high accumulation of total soluble sugars was observed in the plants of both quinoa cultivars raised from cold-stressed seeds. Cultivar V_1_ showed a uniform response in sugar accumulation to both foliar and pre-sowing proline treatments, whereas foliar treatment gave more significant results in cv. V_2_ as compared to the other cultivar (Figure 3).

A marked increase in total free amino acids in both quinoa cultivars was observed due to low-temperature stress applied as seed treatment. However, foliar and pre-sowing proline applications significantly (*P* ≤ 0.01) increased the total free amino acids in both quinoa cultivars. Cultivar V_2_ was better at the rate of 50 mM proline applied as a foliage spray, whereas cultivar V_1_ accumulated higher amounts of amino acids at 50 mM proline applied as a pre-sowing treatment (Figure 3).

## 4. Discussion

Biochemical analyses of plants under a variety of abiotic stresses reflect the fact that plants accumulate compatible metabolites significantly, which effectively protects the plants under stress [47,48]. As compared to the other osmoprotectants, proline accumulates in sufficient amounts and interferes with the plant defensive metabolic system by scavenging reactive oxygen species so as to improve plant growth and metabolism [16]. The stress-sensitive plants incapable of producing this amino acid remain usually intolerant to ecological stresses [16]. Similarly, it is evident that the exogenous use of proline provides relief against stress injuries [36]. It has been observed that treatment of this amino acid not only enhanced adaptive plant metabolism but also accelerated nitrogen absorption and its assimilation [49]. In the present study, the shoot fresh and dry weights of quinoa plants decreased significantly, but no significant change was observed on the root fresh and dry weights of plants raised from cold-stressed seeds. Both modes (foliar and pre-sowing) of proline application significantly improved the shoot and root fresh and dry weights of both quinoa plants. Similarly, in the case of plant height, low-temperature stress applied as a seed treatment significantly reduced shoot length, but it did not affect the root length. Both modes of proline application significantly enhanced the shoot and root lengths of quinoa plants raised from low-temperature treated seeds. Similar effects on growth attributes have already been reported in maize [25], quinoa [50] and faba bean [51] under drought stress, rice [52] and fenugreek plant [53] under salinity stress. These findings show that the application of proline ameliorates the adverse effects of abiotic stresses, perhaps by modulating cell division and cell elongation so as to produce a high plant biomass and vigor. Exogenously applied proline acts as an important nutrient, which could be beneficial for stress tolerance as well as enhancing the activities/levels of enzymatic and non-enzymatic antioxidants [54,55].

Prolonged stress conditions are believed to cause an impairment in the accumulation of photosynthetic pigments such as chlorophyll in plants [56], as a result of which photosynthetic events such as the electron transport chain and conversion of light energy into metabolic energy undergo alteration [57,58]. In the current study, plants raised from cold-treated seeds showed reduced chlorophyll *a* and *b*, total chlorophyll, and carotenoids in both quinoa cultivars, whereas the application of proline significantly improved all these pigments. These results are in line with those of Elewa, et al. [50], in which an increase in chlorophyll pigments was observed due to proline spray at the rate of 12.5 and 25 mM to quinoa plants exposed to drought stress. A reduction in photosynthetic pigments could have been due to stress-induced impairment in pigment synthesis [59]. In sunflower plants, Sadak and Mostafa [60] found a significant enhancement in chlorophyll *a* and *b* and carotenoids due to proline (2.5. 5.0 and 7.5 mM) application as a pre-sowing treatment under drought stress. Similar findings were reported by Ali, et al. [51] in faba bean plants and Hasanuzzaman, et al. [61] in rice plants under salt stress. The degradation of chloroplast and photosynthetic pigments due to stress conditions has a severe impact on the accumulation of carbon assimilates [51]. 

Under stress conditions, plants counteract the stress-induced adversaries by accumulating compatible osmoprotectants such as glycine betaine and proline etc., which play a key role in regulating osmotica accumulation in plants [19]. The external use of such potential protectants ameliorates the adverse effects of oxidative stress by activating the antioxidant defense system and the maintenance of osmotic homeostasis, turgor potential, and water relations [62]. Likewise, in the present study, chilling stress applied as a seed treatment increased endogenous concentrations of proline and glycine betaine in both quinoa cultivars. The exogenous application of proline also posed a positive effect in enhancing the accumulation of both these osmoprotectants in plants raised from cold-treated seeds. These findings are consistent with those of Abd Elhamid, et al. [53], in which the endogenous level of proline was found to be improved upon proline external application in saline-stressed *Phaseolus vulgaris*. In barley seedlings, exogenous application of proline improved in vivo levels of proline [63], whereas in rice plants, intracellular proline levels were increased upon a topical spray of proline (5 mM) under saline stress [61]. In view of the above results, it can be suggested that proline is an effective protectant to make plants robust against abiotic stress adversity.

Ascorbic acid acts as an antioxidant, which can preserve plant photosynthesis and aerobic metabolic activities, which are otherwise deteriorated during oxidative stress [64]. Chilling stress applied as a pre-sowing seed treatment caused a significant increase in ascorbic acid (AsA) concentration in both quinoa cultivars. However, foliar-applied proline proved to be more efficient in promoting ascorbic acid levels than the seed priming treatment in quinoa plants. Earlier, Sakr, et al. [65] reported that a foliar spray of proline at 200 mg L^−1^ enhanced the levels of ascorbic acid under salinity stress in canola plants. Similarly, Abdelhamid, et al. [66] reported that the treatment of 5.0 mM proline elevated AsA levels in *Phaseolus vulgaris* under saline stress

Plants exposed to stressful environments experience enhanced lipid peroxidation and malondialdehyde contents. High MDA contents are generally considered to be a potential indicator of oxidative stress in plants [67,68]. High production of H_2_O_2_ as well as MDA is also one of the toxic effects of oxidative stress [69]. In the present study, chilling stress applied as a pre-sowing treatment had a significant effect on H_2_O_2_ concentration and levels of MDA in both quinoa cultivars. Both foliar and pre-sowing treatments of proline did not play a significant role in lowering H_2_O_2_ levels, but they reduced the MDA contents in both quinoa cultivars. Addressing this concern, a previous study by Hasanuzzaman, et al. [61] showed that an external application of proline reduced H_2_O_2_ and MDA levels in rice plants under salinity stress by lowering the oxidative defense system. These results are in agreement with those of Ali, et al. [51], in which it was reported that drought stress enhanced the malondialdehyde in faba bean plants. Generally, it is believed that proline treatment lowers the production of free radicals and subsequently retards lipid peroxidation [36,70]. 

Plants upon exposure to stress conditions usually experience adaptive physiological changes. Under adverse stress conditions, the accumulation of antioxidants is an important phenomenon. High activities of antioxidant enzymes such as catalase, superoxide dismutase and peroxidase play an important role in ameliorating stress injuries caused by reactive oxygen species [71]. The present study showed that cold-stress conditions applied as a pre-sowing seed treatment significantly increased the activities of peroxidase enzyme but remained non-significant for catalase and superoxide dismutase activities. The treatments of proline as seed soaking and as a foliar spray improved the activities of CAT, POD and SOD in plants of both quinoa cultivars raised from low-temperature treated seeds, which indicated that proline may be involved in the oxidative defense system. These findings are in line with some previous reports conducted under different abiotic stresses; e.g., Osman [71] reported that the application of proline to pea plants under drought stress showed improved activities of catalase and ascorbate peroxidase. Similar results were reported by Elewa, et al. [50] in quinoa, faba bean [51], and barley [63]. The enhancement of antioxidant enzymes could be effective in improving the growth and development of most plants under stress conditions [72]. 

The intrinsic total soluble phenolics increased in quinoa plants in response to cold stress applied as a seed treatment. The external application of proline was observed to be effective in further improving the phenolic levels. These results are in agreement with those of Elewa, et al. [50] in quinoa and Abd Elhamid, et al. [53] in tomato. It could be possible that phenolics function in conjunction with other potential antioxidants to counteract ROS [73]. 

To survive under stress, the accumulation of osmoregulators as well as oxidative scavengers are important defensive features of plants. In this study, cold stress applied as a pre-sowing treatment caused a marked accumulation of total soluble sugars in plants of both quinoa cultivars. Overall, both modes of proline application significantly improved total soluble sugars in plants raised from seeds treated with cold. In contrast, Ben Ahmed, et al. [74] reported a decrease in sugar levels in proline-treated olive plants under salinity stress. Recently, Zali and Ehsanzadeh [75] have reported an enhanced accumulation of sugars in fennel plants exposed to drought stress. It is believed that sugar levels during stress may neutralize the reactive oxygen species and help maintain the turgidity of cells and the integrity of cellular membranes [76].

## 5. Conclusions

In conclusion, chilling stress adversely affected the plant growth and metabolism of quinoa plants. Furthermore, the exogenous application of proline was found to be beneficial for improving growth, chlorophyll *a*, carotenoids, glycine betaine, ascorbic acid, the activities of antioxidant enzymes, and in lowering the hydrogen peroxide and malondialdehyde contents in both quinoa cultivars. As a whole, the exogenous application of proline as a foliar spray or seed treatment were more beneficial than the other treatments used.

## Figures and Tables

**Table 1:** Analyses of variance data for different physio-biochemical attributes of chilling stressed quinoa (*Chenopodium quinoa*) plants subjected to varying (25 and 50 mM) levels of foliar-applied proline. SOD: superoxide dismutase.

Source of Variation	df	Shoot Fresh Weight	Shoot Dry Weight	Root Fresh Weight	Root Dry Weight	Shoot Length	Root Length
Cultivars (Cv)	1	0.132 ns	0.0002 ns	0.0005.8 ns	0.00005 ns	0.16 ns	0.0011 ns
Chilling Stress (CS)	1	1.311 ***	0.0196 ***	0.0072 ***	0.0001.1 *	11.33 *	207.36 ***
Foliar Spray (FS)	2	0.273 ns	0.0034 ns	0.0015 ***	0.0043 *	5.94 ns	54.5 **
Cv x CS	1	0.409 *	0.00024 ns	0.00054 ns	0.00002 ns	2.66 ns	34.41 *
Cv x FS	2	0.044 ns	0.0005.5 ns	0.000009 ns	0.00003 ns	1.05 ns	1.02 ns
CS x FS	2	0.014 ns	0.0006 ns	0.00002 ns	0.00002 ns	1.48 ns	0.955 ns
Cv x CS x FS	2	0.036 ns	0.0002.6 ns	0.0002 ns	0.000002 ns	0.564 ns	2.62 ns
Error	24			0.0001	0.00002	1.82	7.683
		**Chl. *a***	**Chl. *b***	**Chl. a/b**	**Total Chl.**	**Carotenoids**	**Proline**
Cultivars (Cv)	1	0.019 ns	0.0003 ns	0.174 ns	0.014 ns	0.0001 ns	79.59 ns
Chilling Stress (CS)	1	0.232 **	0.104 ***	0.837 *	0.648 ***	0.0876 ***	1070.26 ***
Foliar Spray (FS)	2	0.184 **	0.045 ***	0.308 ns	0.407 ***	0.0245 **	244.5 **
Cv x CS	1	0.169 *	0.0097 ns	0.193 ns	0.26 **	0.001 ns	5.93 ns
Cv x FS	2	0.0352 ns	0.0093 ns	1.107 **	0.0088 ns	0.00005 ns	15.75 ns
CS x FS	2	0.0269 ns	0.0045 ns	0.0308 ns	0.052 ns	0.0039 ns	40.36 ns
Cv x CS x FS	2	0.0214 ns	0.00563 ns	0.52 ns	0.0165 ns	0.000065 ns	4.64 ns
Error	24	0.0269	0.0029	0.185	0.9332	0.0033	35.04
		**Glycine Betaine**	**Ascorbic Acid**	**Hydrogen Peroxide**	**MDA**	**Catalase**	**Peroxidase**
Cultivars (Cv)	1	6553.30 ***	4.54 ns	1463.05 ns	2.9221e-4 ns	1.24 ***	0.0035 ns
Chilling Stress (CS)	1	288.79 ns	80.14 ***	20928.4 **	116.7 ***	0.013 ns	0.030 *
Foliar Spray (FS)	2	259.63 ns	50.41 ***	13452.4 **	24.30 ***	0.059 *	0.0117 ns
Cv x CS	1	2106.43 *	0.978 ns	28.44 ns	18.48 *	0.182 ns	0.0087 ns
Cv x FS	2	175.61 ns	3.16 ns	2696.2 ns	1.54 ns	0.0033 ns	0.0023 ns
CS x FS	2	648.77 ns	18.22 ns	1711.5 ns	0.449 ns	0.0124 ns	0.0001 ns
Cv x CS x FS	2	710.5 ns	1.37 ns	464.9 ns	2.12 ns	0.0041 ns	0.0009 ns
Error	24	274.01	2.414	2299.2	2.45	0.0155	0.0042
		**SOD**	**Total Soluble Sugars**	**Total Phenolics**	**Total Free Amino Acids**		
Cultivars (Cv)	1	1.104 ns	62.93 *	39.27 ns	0.0262 **		
Chilling Stress (CS)	1	7.02 *	228.01 ***	686.44 ***	0.0162 *		
Foliar Spray (FS)	2	8.909 *	114.3 ***	122.06 **	0.0078 *		
Cv x CS	1	4.867 ns	0.694 ns	78.91 ns	0.0008 ns		
Cv x FS	2	2.111 ns	17.3 ns	15.57 ns	0.0001 ns		
CS x FS	2	2.91 ns	18.73 ns	34.22 ns	0.0006 ns		
Cv x CS x FS	2	3.76 ns	54.72 ns	42.4 ns	0.0012 ns		
Error	24	1.642	11.51	18.57	0.002		

ns = non-significant; *, ** and *** = significant at 0.05, 0.01 and 0.001 levels, respectively.

**Table 2:** Analyses of variance of data for different physio-biochemical attributes of quinoa (*Chenopodium quinoa*) plants raised from seeds treated with varying (25 and 50 mM) levels of proline and chilling stress.

Source of Variation	df	Shoot Fresh Weight Shoot Fresh Weight	Shoot Dry Weight	Root Fresh Weight	Root Dry Weight	Shoot Length	Root Length
Cultivars (Cv)	1	0.558 *	0.016 ***	0.0027 **	0.000006 ns	19.65 ***	6.33 ns
Chilling Stress (CS)	1	3.091 ***	0.0113 **	0.0047 ***	0.00001 *	2.89 ns	107.8 ***
Presowing (Pre)	2	1.216 ***	0.0052 *	0.0038 ***	0.00005 ns	10.11 ns	69.21 ***
Cv x CS	1	0.098 ns	0.004 ns	0.00005 ns	0.0000009 ns	0.027 ns	26.18 *
Cv x Pre	2	0.32 *	0.0008 ns	0.00006 ns	0.000000015 ns	1.66 ns	8.07 ns
CS x Pre	2	0.0261 ns	0.00005 ns	0.000008 ns	0.000001 ns	0.663 ns	2.09 ns
Cv x CS x Pre	2	0.0305 ns	0.0005 ns	0.00002 ns	0.0000007 ns	0.231 ns	15.93 ns
Error	24	0.0834	0.00113	0.000002	0.00001	1.394	4.87
		**Chl. *a***	**Chl. *b***	**Chl. a/b**	**Total Chl.**	**Carotenoids**	**Proline**
Cultivars (Cv)	1	1.23 ***	0.777 ***	34.58 ***	0.0532 ns	0.015 *	445.49 *
Chilling Stress (CS)	1	0.703 ***	0.951 ***	5.49 *	3.29 ***	0.0006 ns	611.02 **
Presowing (Pre)	2	0.273 **	0.223 ***	1.80 ns	0.933 ***	0.011 **	909.4 ***
Cv x CS	1	0.559 ***	0.462 ***	0.497 ns	2.04 ***	0.0054 ns	5.33 ns
Cv x Pre	2	0.159 *	0.052 *	2.83 *	0.039 ns	0.00001 ns	3.88 ns
CS x Pre	2	0.044 ns	0.060 *	0.136 ns	0.205 **	0.000008 ns	120.9 ns
Cv x CS x Pre	2	0.069 ns	0.0809 **	2.46 ns	0.0035 ns	0.0009 ns	262.5 *
Error	24	0.0342	0.0131	0.772	0.024	0.002	72.99
		**Glycine Betaine**	**Ascorbic Acid**	**Hydrogen Peroxide**	**MDA**	**Catalase**	**Peroxidase**
Cultivars (Cv)	1	633.52 **	28.13 **	1158762.5 ***	26.82 ns	0.142 **	0.00002 ns
Chilling Stress (CS)	1	1386.6 ***	19.09 *	317579.2 **	266.2 ***	0.049 ns	0.199 ***
Presowing (Pre)	2	414.3 **	49.14 ***	67016.8 ns	34.64 ns	0.050 ns	0.0302 **
Cv x CS	1	424.9 *	1.98 ns	21085.4 ns	3.5 ns	0.019 ns	0.07 **
Cv x Pre	2	6.90 ns	1.392 ns	10304.3 ns	19.68 ns	0.0015 ns	0.00001 ns
CS x Pre	2	0.648 ns	15.92 *	75875.6 ns	8.54 ns	0.0047 ns	0.0104 ns
Cv x CS x Pre	2	27.11 ns	12.34 *	7958.5 ns	0.75 ns	0.0079 ns	0.00008 ns
Error	24	58.01	3.04	28230.2	10.89	0.0152	0.00008
		**SOD**	**Total Soluble Sugars**	**Total Phenolics**	**Total Free Amino Acids**		
Cultivars (Cv)	1	22.35 ***	244.92 *	570.6 ***	0.003 ns		
Chilling Stress (CS)	1	18.34 **	121.4 ns	1348.4 ***	0.022 **		
Presowing (Pre)	2	2.14 ns	72.27 ns	41.28 ns	0.005 ns		
Cv x CS	1	0.292 ns	6.33 ns	0.307 ns	0.0037 ns		
Cv x Pre	2	0.782 ns	29.98 ns	1.195 ns	0.00001 ns		
CS x Pre	2	0.682 ns	93.94 ns	12.003 ns	0.0002 ns		
Cv x CS x Pre	2	0.206 ns	22.75 ns	0.284 ns	0.00008 ns		
Error	24	1.56	36.22	18.87	0.0019		

ns = non-significant; *, ** and *** = significant at 0.05, 0.01 and 0.001 levels, respectively.

## Supplementary Materials

The following are available online at https://www.mdpi.com/2223-7747/8/12/588/s1, Figure S1: Overview of both quinoa cultivars V1 (A) and V2 (B) subjected to exogenously applied proline and cold stress conditions.
