# Supplementary material for: Seed Pretreatment and Foliar Application of Proline Regulate Morphological, Physio-Biochemical Processes and Activity of Antioxidant Enzymes in Plants of Two Cultivars of Quinoa (Chenopodium quinoa Willd.)
Source: Plants (Basel). 2019 Dec 10;8(12):588. doi: 10.3390/plants8120588 (PMC6963800; doi:10.3390/plants8120588)
Supplement: Supplementary file 1 [file plants-08-00588-s001.pdf]

plants

MDPI

A

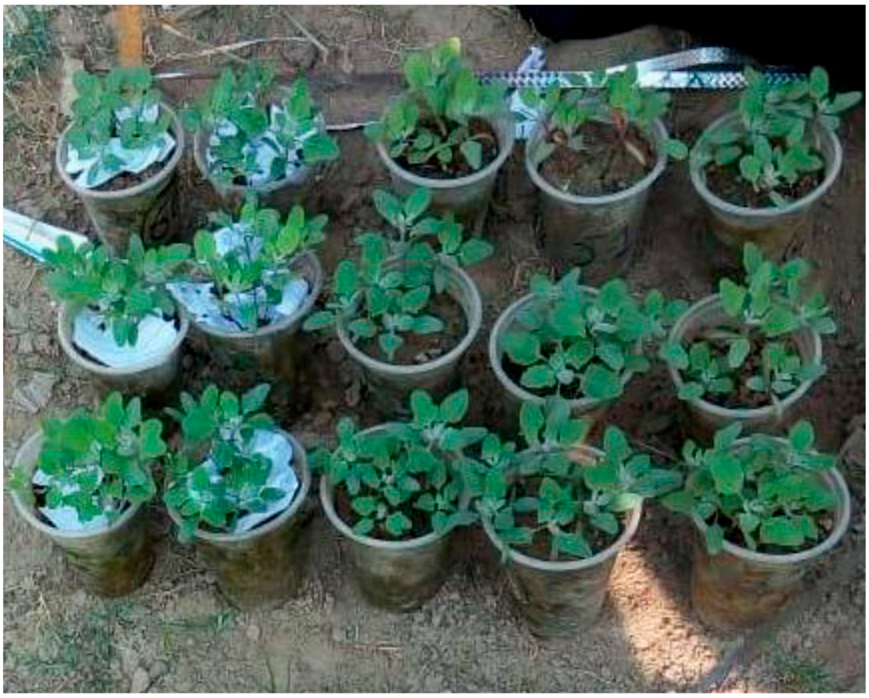

B

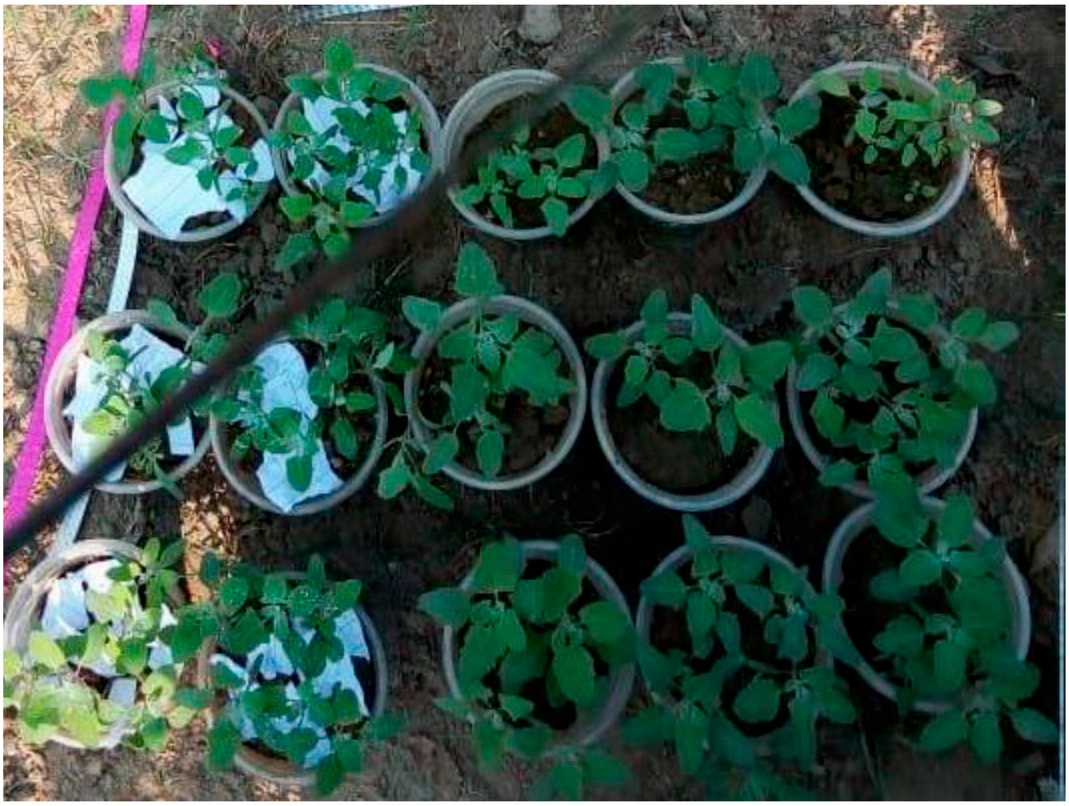

Figure 4. Overview of both quinoa cultivars V1 (**A**) and V2 (**B**) subjected to exogenously applied proline and cold stress conditions.
